# Unravelling the mechanisms of vibrational relaxation in solution†
†All experimental data are archived in the University of Bristol's Research Data Storage Facility (DOI: 10.5523/bris.2vk036f35m5aq2dnlb79c0wcsh).
‡
‡Electronic supplementary information (ESI) available: Further discussion of spectral lineshapes, concentration dependence of transient absorption data, theoretical calculations, IR-pump IR-probe spectra, transient absorption spectra including animation of spectra. See DOI: 10.1039/c6sc05234g



**DOI:** 10.1039/c6sc05234g

**Published:** 2017-02-10

**Authors:** Michael P. Grubb, Philip M. Coulter, Hugo J. B. Marroux, Andrew J. Orr-Ewing, Michael N. R. Ashfold

**Affiliations:** a School of Chemistry , University of Bristol , Cantock's Close , Bristol BS8 1TS , UK . Email: a.orr-ewing@bristol.ac.uk ; Email: mike.ashfold@bristol.ac.uk; b Department of Chemistry , Fort Lewis College , Durango , Colorado 81301 , USA

## Abstract

We present a systematic study of the mode-specific vibrational relaxation of NO_2_ in six weakly-interacting solvents (perfluorohexane, perfluoromethylcyclohexane, perfluorodecalin, carbon tetrachloride, chloroform, and d-chloroform), chosen to elucidate the dominant energy transfer mechanisms in the solution phase. Broadband transient vibrational absorption spectroscopy has allowed us to extract quantum state-resolved relaxation dynamics of the two distinct NO_2_ fragments produced from the 340 nm photolysis of N_2_O_4_ → NO_2_(X) + NO_2_(A) and their separate paths to thermal equilibrium. Distinct relaxation pathways are observed for the NO_2_ bending and stretching modes, even at energies as high as 7000 cm^–1^ above the potential minimum. Vibrational energy transfer is governed by different interaction mechanisms in the various solvent environments, and proceeds with timescales ranging from 20–1100 ps. NO_2_ relaxation rates in the perfluorocarbon solvents are identical despite differences in acceptor mode state densities, infrared absorption cross sections, and local solvent structure. Vibrational energy is shown to be transferred to non-vibrational solvent degrees of freedom (V-T) through impulsive collisions with the perfluorocarbon molecules. Conversely, NO_2_ relaxation in chlorinated solvents is reliant on vibrational resonances (V-V) while V-T energy transfer is inefficient and thermal excitation of the surrounding solvent molecules inhibits faster vibrational relaxation through direct complexation. Intramolecular vibrational redistribution allows the symmetric stretch of NO_2_ to act as a gateway for antisymmetric stretch energy to exit the molecule. This study establishes an unprecedented level of detail for the cooling dynamics of a solvated small molecule, and provides a benchmark system for future theoretical studies of vibrational relaxation processes in solution.

## Abstract 2

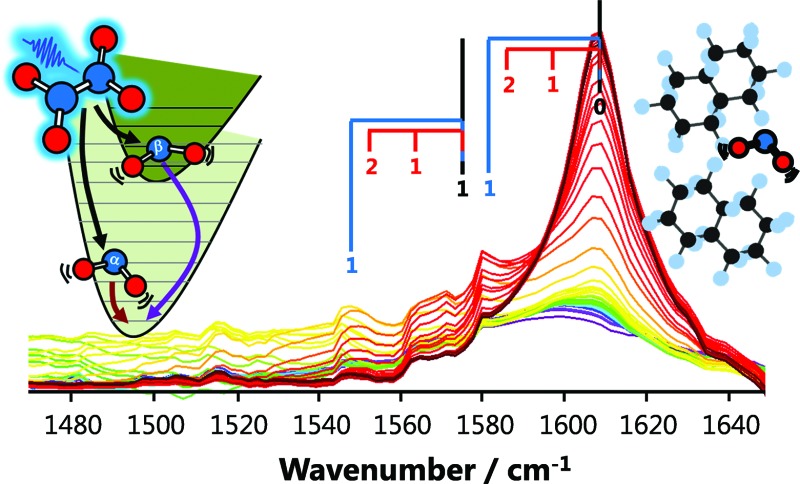
Time resolved vibrational cooling towards equilibrium in perfluorinated and chlorinated solvents provides detailed insights into the transfer of energy between solute and solvent molecules.

## Introduction

Chemical reactions in solution are commonplace in living organisms, the natural environment, and in synthetic and industrial processes. The rates of these reactions can be strongly dependent upon the internal energy of the reactants, and hence upon the mechanisms and rates with which energy flows between solute and solvent molecules. The solute molecules are continuously exposed to forces from the surrounding solvent, and the interplay of these solute–solvent interactions can stabilise or destabilise reactive intermediates, and control the exchange of energy and reaction mechanisms. Measurements of the flow of energy from a solute to the solvent probe the intermolecular interactions, but are challenging given their complexity and frequency.

The timescale of vibrational energy relaxation in solution can range from a few picoseconds up to several seconds in some instances, and a reliably predictive theoretical model for these widely varying relaxation processes is still out of reach.^1–3^ Here we present a systematic study of the (mode-specific) vibrational cooling dynamics of energized solute molecules (NO_2_) in six simple solvents, chosen to allow systematic examination of the mechanisms by which energy relaxation occurs in solution, and to distinguish between competing solute-to-solvent energy transfer pathways.

Highly vibrationally excited NO_2_ can be generated promptly from the photolysis of N_2_O_4_, which dissolves readily in all of the solvents used and is formed by bubbling in gaseous NO_2_. Photolysis at 340 nm generates two NO_2_ molecules: one (α) in the ground X^2^A_1_ state, the other (β) in the first excited A^2^B_2_ state.^4^ 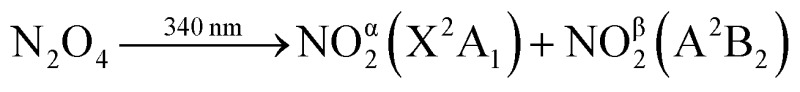


The NOα2 fragment is produced with modest vibrational excitation. The NOβ2 fragment is formed with both electronic and vibrational excitation, but rapidly internally converts to high vibrational levels of the ground state. This second pathway provides a source of highly vibrationally excited non-thermal NO_2_.

Vibrational energy transfer is generally discussed in terms of donating and accepting modes. Two broad categories describe the nature of the donating–accepting pair interaction in the previous literature: solute vibration to solvent vibration energy transfer (V-V) and solute vibration to solvent translation/rotation/libration transfer (V-T).^1,5–8^ Three primary energy transfer mechanisms are useful for the current discussion. These mechanisms for solute vibrational energy relaxation to the solvent are identified through their interaction type:

(1) Impulsive coupling (V-T and/or V-V): energy transfer through short-range repulsive interactions. This coupling is more efficient for ‘hard’ collisions.^9,10^

(2) Anharmonic coupling (V-V): vibrational energy is transferred *via* short-range attractive interactions. This mechanism requires some degree of solute–solvent complexation, most notably through hydrogen bonding.^11^

(3) Förster coupling (V-V): vibrational energy is transferred through transition dipole–dipole interactions, as a virtual photon. Förster coupling requires spectral overlap of the solute infrared (IR) emission and solvent IR absorption spectra, and decays with increasing solute–solvent distance (*R*) with *R* ^–6^ dependence.^12–14^

The solvents used in the current studies were chosen to enhance specific energy transfer mechanisms. The three perfluorinated solvent molecules – perfluorohexane, perfluoromethylcyclohexane, and perfluorodecalin – have negligible dipole moments and minimal polarizability, and thus experience only weak attractive interactions with solute molecules. However, each solvent possesses unique vibrational frequencies and bulk/molecular solvent structures, which can affect Förster and impulsive coupling mechanisms. Perfluorodecalin contains 1.4 times as many internal degrees of freedom as the other perfluorinated solvents, and thus should accept energy faster in any purely statistical picture. Carbon tetrachloride (CCl_4_) also has no permanent molecular dipole moment, but is significantly more polarizable than the perfluorocarbons (PFCs) and thus experiences stronger dispersion interactions with the solute. Chloroform (CHCl_3_) is similar to CCl_4_ in terms of vibrational modes and dispersion interaction strength, but possesses a permanent dipole moment (∼1.04 D)^15^ which facilitates stronger complexation with a polar solute molecule. Deuterated chloroform (CDCl_3_) is chemically identical to CHCl_3_, but some of its vibrational frequencies are shifted by the heavier deuterium atom. The effects of these different solute–solvent interactions are clearly distinguished in our ultrafast time-resolved IR absorption measurements of the vibrational cooling of internally hot NO_2_ in the various solvents.

## Results and discussion

**Fig. 1:**
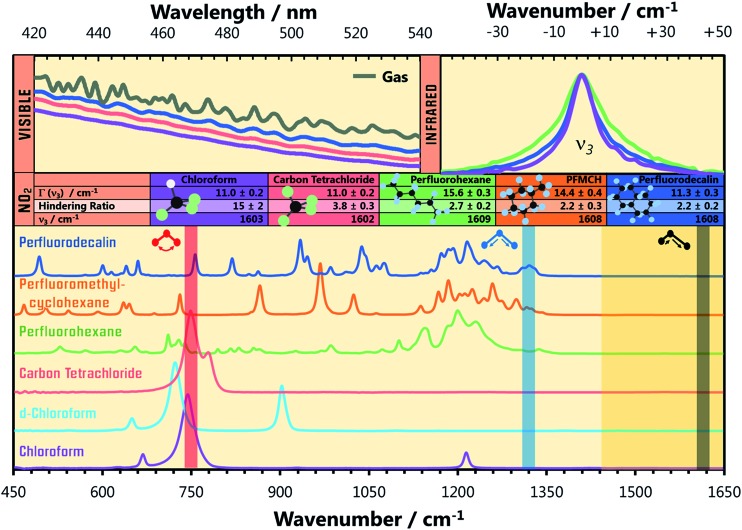
IR absorption spectra (bottom) of the solvents used in the present experiments. The translucent vertical bands overlaid on the solvent spectra indicate the wavenumbers of the three vibrational modes of NO_2_, and highlight potential resonant vibrational modes of the solvent. The IR absorption spectra of the NO_2_ (*ν*_3_ = 1 ← 0) fundamental transition in perfluorohexane, perfluorodecalin, and chloroform are shown (top right) with their central wavenumbers shifted to the same value for the sake of comparison. The true central wavenumbers of this transition (*ν*_3_), the Lorentzian linewidth *Γ*, and ratio of rotationally hindered to unhindered spectral contributions (see ESI‡) are provided in the table. The visible spectra of NO_2_ in the gas phase, in perfluorodecalin, carbon tetrachloride, and in chloroform (top left) demonstrate the increasing degree of inhomogeneous broadening induced by solute–solvent interactions in this sequence of environments.

Steady state electronic absorption spectra of NO_2_ dissolved in perfluorodecalin (which was indistinguishable from the spectra of NO_2_ dissolved in perfluorohexane and perfluoromethylcyclohexane), CCl_4_, and CHCl_3_ are shown in Fig. 1 alongside the gas phase spectrum (at 1 atm). The gas-phase vibronic structure of the NO_2_ is partially or completely lost through inhomogeneous broadening by solvent interactions, which can be modelled by convoluting the gas phase spectrum with a Gaussian function. The degree of spectral broadening provides valuable information about the local solvation environment of the NO_2_ in these solutions. The solution phase electronic spectra of NO_2_ show an increasing Gaussian linewidth (FWHM) with the intermolecular interaction strength of the solvent, from PFCs (∼230 cm^–1^), to CCl_4_ (∼500 cm^–1^), and CHCl_3_ (∼600 cm^–1^).

The shapes of individual bands in the infrared (IR) vibrational spectrum also inform about the local solvation of the NO_2_. Spectra of the ∼1610 cm^–1^ transition of NO_2_ in perfluorohexane, perfluorodecalin, and chloroform are shown in Fig. 1. Each spectrum has been shifted to the same central transition wavenumber for better comparison of the lineshapes, but the true transition wavenumbers (*ν*_3_ in Fig. 1) are provided in the accompanying table. All of the transitions are Lorentzian broadened, indicating that homogeneous broadening dominates the line shape. The Lorentzian linewidth *Γ* reflects the magnitude and timescale of solvent-induced perturbations to the solute transition frequency. The similarity of these linewidths and the solvent-induced band shifts (Fig. 1) indicates comparable solute–solvent interaction potentials for the three PFC solvents, albeit with some subtle differences in the local solvation environments of the NO_2_. Significant signal contributions were observed in the wings of the spectral band that cannot be captured by a single Lorentzian function, but can be fit when some P/R branch rotational character is included in the lineshape. We recently established that (small molecule) solute rotation is relatively unhindered in PFC solutions, leading to the observation of significant rotational band structure in the absorption spectrum.^16^ The degree to which the solute rotation is hindered in each solvent provides further insights about the local solute environment. Further discussion of this effect is provided in the ESI.‡

**Fig. 2:**
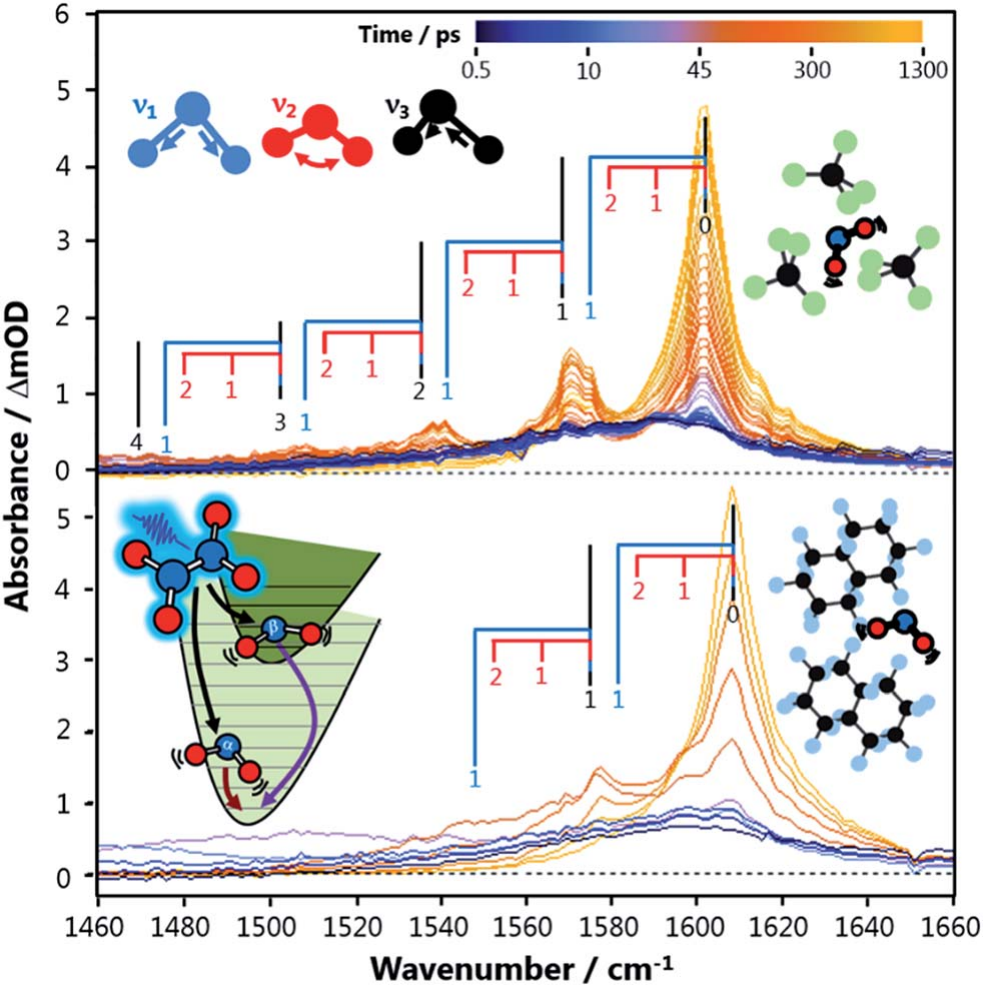
Transient vibrational absorption spectra of the antisymmetric stretch vibration (*ν*_3_) of NO_2_ X^2^A_1_ produced from N_2_O_4_ photolysis at 340 nm in a liquid solution of CCl_4_ (top) and perfluorodecalin (bottom). Excitation of the symmetric stretch (*ν*_1_) and bend (*ν*_2_) modes can also be observed due to anharmonic coupling with *ν*_3_. The combs identify the absorption bands corresponding to NO_2_ molecules with different degrees of excitation in the three vibrational modes. The inset shows schematically how N_2_O_4_ dissociation produces two NO_2_ molecules with different electronic and vibrational character, labelled as NOα2 and NOβ2 in the main text.

NO_2_ vibration is described by three normal modes: the symmetric stretch (*ν*_1_ = 1320 cm^–1^), bend (*ν*_2_ = 750 cm^–1^), and antisymmetric stretch (*ν*_3_ = 1617 cm^–1^).^17^ The vibrational state-populations of both NO_2_ fragments from N_2_O_4_ photolysis were tracked as a function of time through the IR absorption of the *ν*_3_ transitions with sub-picosecond temporal resolution, using broadband Transient Vibrational Absorption Spectroscopy. While the *ν*_3_ mode is probed directly, the *ν*_2_ and *ν*_1_ state populations can also be observed through their anharmonic coupling to the antisymmetric stretch (*x*_33_ = –16.6 cm^–1^, *x*_23_ = –11.1 cm^–1^, *x*_13_ = –26.9 cm^–1^).^17^ The way this coupling reveals itself in the vibrational spectra is shown by the superimposed combs in Fig. 2.

Fig. 2 shows transient vibrational spectra of the NO_2_ antisymmetric stretch as a function of pump–probe delay time after the photolysis of N_2_O_4_ at 340 nm in CCl_4_ and perfluorodecalin solutions. The initial internal energy of the NOα2 fragment can be clearly observed from the breadth of the dark blue 0.5 ps trace, and is well described by a 1350 K Boltzmann vibrational distribution across all three vibrational modes. The initial distribution is not dependent on the choice of solvent, indicating the solvent environment has little effect on the N_2_O_4_ dissociation dynamics. The NOβ2 fragment is not observed until after it internally converts to high vibrational levels of the X^2^A_1_ state and subsequently cools to *ν*_3_ ≤ 7 (levels with *ν*_3_ ≤ 4 are revealed in the spectral region presented in Fig. 2). This delayed observation is especially evident in the perfluorinated solvents, where the signal from this fragment moves into the spectral window from the low wavenumber side as a localized envelope of absorption signal (see ESI‡ for animated transient spectra in all solvents, and for spectra showing the earlier time cooling among higher vibrational levels in the region to lower wavenumber).

### Vibrational relaxation in the X^2^A_1_ state

After pump–probe time delays Δ*t* > 150 ps, both NO_2_ fragments are sufficiently vibrationally relaxed that their combined spectral contributions can be described by a Boltzmann distribution of NO_2_ vibrational state populations. In all solvents, the spectra reveal that the populations of the *ν*_1_ and *ν*_3_ vibrational modes evolve concurrently, indicating that intramolecular vibrational redistribution (IVR) between the two stretching modes is faster than the dissipation of vibrational energy to the solvent. Fig. 2 shows that the signal attributable to population in *ν*_2_ = 1 at 1590 cm^–1^ in CCl_4_ rapidly disappears; however, it persists as a shoulder on the *ν*_3_ = 1 ← 0 absorption feature in perfluorodecalin for 100 s of ps demonstrating that population in the NO_2_ bending mode evolves on different timescales in different solvents. The populations of the various vibrational levels within each mode are always well-described by a Boltzmann distribution of states after Δ*t* > 150 ps, and all subsequent Δ*t* spectra could be reproduced with a simple model where the vibrational temperatures of the *ν*_1,3_ and *ν*_2_ modes were the only variables. The thermalization within each mode provides an important constraint when extracting state populations from the transient spectra, which are otherwise underdetermined for transitions in the 1480–1560 cm^–1^ spectral range where the density of spectral transitions becomes large.

Each transient spectrum after Δ*t* = 150 ps was simulated with a best-fit model spectrum, in which the only adjustable parameters were the Boltzmann temperatures for the stretching and bending modes. The anharmonically coupled transition wavenumbers were calculated under the assumption that the gas-phase *x*_e_ values did not change in solution, but the central wavenumber of the *ν*_3_ = 1 ← 0 transition was shifted to match the observed experimental wavenumber in each solvent (Fig. 1). The intensity of each *ν*(*n*_1_, *n*_2_, *n*_3_) → *ν*(*n*_1_, *n*_2_, *n*_3_ + 1) transition (with *n*_1_, *n*_2_, *n*_3_ denoting numbers of quanta of the three modes) was set to depend on the difference in population between the *ν*(*n*_1_, *n*_2_, *n*_3_) and *ν*(*n*_1_, *n*_2_, *n*_3_ + 1) states, to include the balance of absorption and stimulated emission. Each transition intensity was further scaled by a factor of *n*_3_ + 1, to account for the predicted linestrength enhancement from vibrational excitation in a harmonic oscillator.^18^ This manner of calculating the transition strengths accurately predicts an integrated intensity from the simulated fit of the initial spectrum (only NOα2) that is equal to half that of the fully relaxed spectrum at Δ*t* = 1200 ps (by which time the NOα2 + NOβ2 fragments are both contributing to the observed spectrum in equal numbers).

**Fig. 3:**
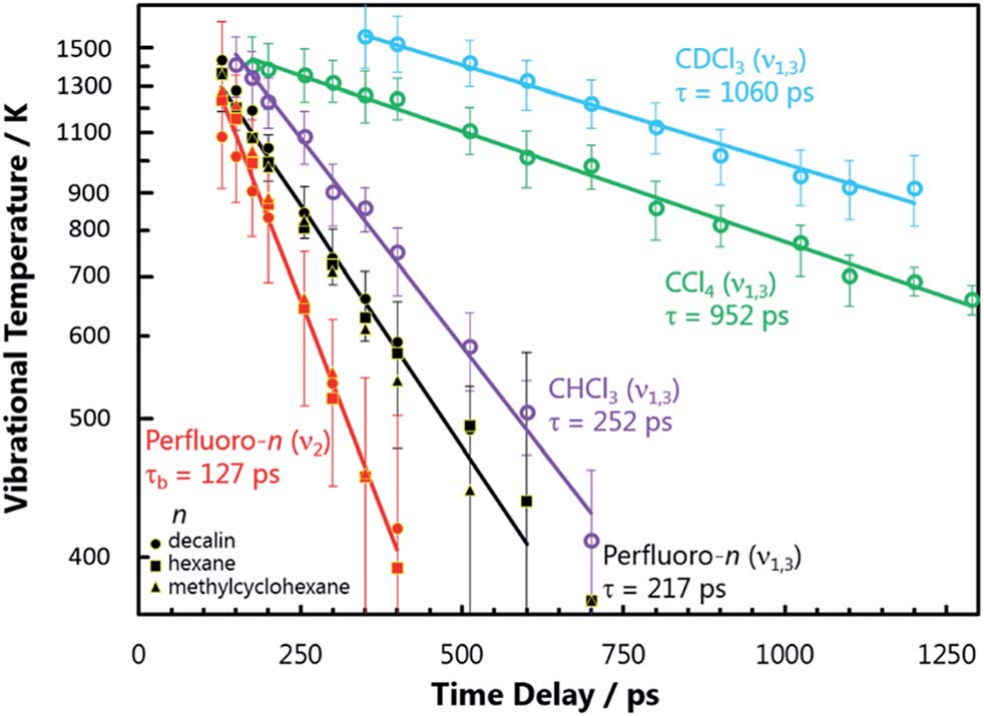
The NO_2_ vibrational temperature (*T*_vib_), extracted from modelling the transient vibrational spectra as a function of pump–probe delay time, plotted using a ln(*T*_vib_ – 293) scale. The slopes of the best-fit lines yield the vibrational temperature relaxation rate coefficients of the modes in parentheses for the indicated solvents. The time constants *τ* are reciprocals of these rate coefficients. NO_2_ vibrational temperatures obtained in each PFC solvent are represented by different symbols, but show the same behaviour and thus are fit with a single line. Only spectra with Δ*t* > 150 ps were used for this analysis, when the vibrational populations have thermalized sufficiently to be well-described by a Boltzmann distribution of states. The error bars represent the range of *T*_vib_ that result in a residual sum of squares between the experimental and model spectrum less than double the best-fit minimum value.

The resulting vibrational temperatures from the best-fit simulations are shown in Fig. 3. The temperature of each vibrational mode in each solvent is observed to cool exponentially in time, as evidenced by the linear slopes of the ln(*T*_vib_ – 293) *vs.* Δ*t* plots. An analysis of the NO_2_ stretch vibrational relaxation as a function of N_2_O_4_ concentration is provided in the ESI.‡ The vibrational temperatures presented in Fig. 3 were observed at N_2_O_4_ concentrations on the order of 200 mM in each solvent, and are found to be only weakly concentration dependent. Hence, semi-quantitative comparisons can be drawn between NO_2_ vibrational temperature relaxation time coefficients in the different solvent environments. NO_2_ bend relaxation in the three chlorinated solvents does not appear on the plot as all excitation in this mode is completely thermalized to 293 K before Δ*t* = 150 ps. A relaxation time constant of *τ*_b_ ∼ 20–50 ps is estimated for this process based on the earlier Δ*t* spectra.

Despite differences in solvent molecular structure, the vibrational relaxation timescales found in the three PFC environments are the same. The significance of the identical NO_2_ bend relaxation time in the three PFC solvents (*τ*_b_ = 127 ps) is first considered. The IR absorption spectra of the three solvents are very different in the 700–750 cm^–1^ range corresponding to intervals between NO_2_ bending levels. The relative integrated absorptions of perfluorohexane, perfluoromethylcyclohexane, and perfluorodecalin respectively are: 1, 0.4, and 0.12 in this wavenumber interval (or 0.67, 1, 0.37 if the range is extended to 650–800 cm^–1^). The large differences in integrated absorption are at odds with the small difference between *ν*_2_ relaxation rates in the three solvents, implying Förster coupling has a negligible effect on this relaxation. Attractive forces in the PFCs are weak, and thus direct anharmonic coupling is not expected to be significant either. Thus we deduce that NO_2_ bend energy is transferred to the perfluorinated solvents primarily through impulsive interactions. It is anticipated that this energy is transferred to non-vibrational degrees of freedom (V-T), since the rate is independent of the solvent's total number of vibrational modes and the density of vibrational states in the 700–750 cm^–1^ range. The energy transfer timescale can be fully explained by the local, repulsive potential energy surfaces of the solute–solvent collisions, which are similar in all three solvents.

Assuming the impulsive model is applicable to all NO_2_ (*ν*) in perfluorinated solvents, the relaxation rates of the higher frequency NO_2_ stretching modes can be predicted using semi-classical theories. Landau–Teller theory, which describes a quantum oscillator in a classical bath, predicts the vibrational relaxation time *τ* to be directly proportional to the frequency of the oscillator *ω*.^1^ Therefore, the ratio of the NO_2_ bend to stretch frequency should give the ratio of their relative relaxation times. The lower *ν*_1_ stretch frequency is used in this analysis because IVR between *ν*_1_ and *ν*_3_ is rapid. This comparison predicts a stretch relaxation time of 225 ps, which is almost exactly what is observed experimentally (*τ*_s_ = 217 ps). So, despite the PFCs having a number of intense IR transitions throughout the 1250–1350 cm^–1^ range, the relaxation time does not appear to be accelerated by these vibrational resonances. Thus, NO_2_ stretch energy is also shown to be lost through impulsive interactions and dissipated to non-vibrational degrees of freedom (V-T) in the PFC solvents.

NO_2_ relaxation in chlorinated solvents is a completely different story. First, it is clear that energy transfer through impulsive interactions is much less efficient than in PFCs. The fastest vibrational energy transfer pathway available for the NO_2_ stretch in CCl_4_ and CDCl_3_ is ∼900 ps, much longer than in the perfluorinated solvents. This is qualitatively expected since the chlorine atoms have a softer repulsive interaction than fluorine atoms. The much faster relaxation of the NO_2_ stretch in CHCl_3_ (*τ*_s_ = 252 ps) compared to CDCl_3_ (*τ*_s_ = 1060 ps), however, is unambiguous evidence of a resonant vibrational enhancement. The C–H bend wavenumber (1220 cm^–1^) in CHCl_3_ is nearly resonance with the NO_2_ *ν*_1_ fundamental transition (1320 cm^–1^), while the C–D bend mode is red-shifted (907 cm^–1^). The resonant effect between NO_2_ and CHCl_3_ occurs even with a vibrational energy mismatch of 100 cm^–1^, and thus coupling to other solvent degrees of freedom may be important in helping bridge this energy gap.

The NO_2_ bend relaxation (20 ps < *τ*_b_ < 50 ps) in chlorinated solvents must also be resonantly enhanced, since this timescale is faster than predicted by the stretch: bend frequency ratio and over four times as fast as in the PFCs. All of the chlorinated solvents have an intense IR absorption from a C–Cl_*n*_ stretching vibration near-resonant with the NO_2_ bend mode in the vicinity of 750 cm^–1^, and thus Förster coupling may be significant. The integrated absorption intensity of this band in CHCl_3_ is nearly 10 times larger than that of the 1210 cm^–1^ band that is near-resonant with the NO_2_ stretch, consistent with the relative *ν*_2_ and *ν*_1,3_ relaxation rates in this solvent and the predictions of Förster theory.

The relaxation of thermally excited NO_2_ formed through the UV photodissociation of N_2_O_4_ can be directly compared to the relaxation timescale of NO_2_ prepared (by IR excitation) with a single quantum of antisymmetric stretch vibration. The majority of the excited population with *ν*_3_ = 1 relaxes with a significantly smaller time constant in CCl_4_ (62 ± 10 ps), CHCl_3_ (23 ± 2 ps) and CDCl_3_ (24 ± 3 ps) than observed for the final *ν*_1,3_ *ν* = 1 → *ν* = 0 step of NO_2_ formed through UV photodissociation of N_2_O_4_ after all higher vibrational levels have relaxed (>1 ns, 180 ps, and >1 ns in the three respective solvents) – see ESI.‡ The apparent discrepancy in these *ν*_1,3_ *ν* = 1 → *ν* = 0 vibrational relaxation times measured in chlorinated solvents is not seen when using the PFC solvents. Instead, the final *ν*_1,3_ *ν* = 1 → *ν* = 0 relaxation of NO_2_ from UV photodissociation of N_2_O_4_ in a PFC solvent occurs with a time constant (120 ± 30 ps) similar to that (89 ± 7 ps) measured for the relaxation of the NO_2_ (*ν*_3_ = 1) molecules prepared by direct IR excitation.

Most of the UV photon energy (29 400 cm^–1^) used to dissociate N_2_O_4_ must be taken up by the solvent molecules in close proximity to the photoexcited solute, resulting in a significant increase in local temperature. Vibrational relaxation is typically accelerated by higher temperature due to increased occupancy of bath modes.^11,19^ However, solute–solvent intermolecular interaction potentials which facilitate solute cooling through anharmonic coupling can be overcome by excess solute or solvent internal energy, thereby increasing the timescale for solute vibrational relaxation.^11^ NO_2_ induces a dipole–dipole interaction with the polarizable CCl_4_, and can interact with the permanent dipole moment of CHCl_3_. Quantum mechanical calculations predict NO_2_–CCl_4_ and NO_2_–CHCl_3_ complexes with intermolecular well depths of a few hundred wavenumbers (cm^–1^) – see ESI.‡ These attractive interactions between NO_2_ and chlorinated solvents are easily disrupted by the thermal energy released during the photodissociation of N_2_O_4_ and subsequent vibrational relaxation of NO_2_ molecules, which inhibits an efficient pathway for solute-to-solvent vibrational energy transfer until the initially hot NO_2_ and its local solvent shell have cooled sufficiently to associate into complexes. The long cooling timescales suggest the energy released by N_2_O_4_ photolysis significantly distorts the local solvent environment, resulting in energy relaxation pathways which are less effective than those that dominate the more weakly perturbing IR-pump measurements. The relaxation of IR-excited NO_2_ is instead controlled by the intermolecular interactions within NO_2_-solvent complexes. Analogous complexes in PFC solvents will only be very weakly bound because of the lower solvent polarizabilities, and are unlikely to contribute significantly to vibrational cooling rates.

### Relaxation of the NOβ2 fragment

**Fig. 4:**
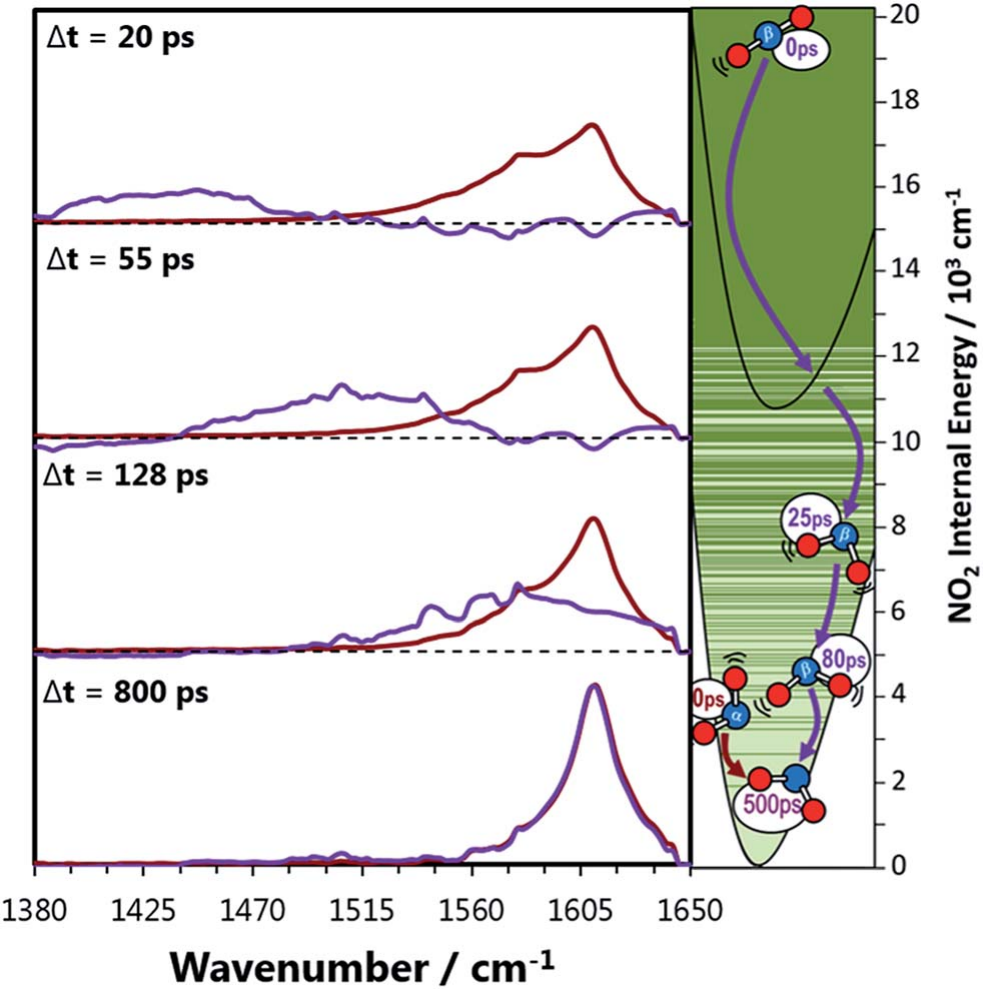
Decomposed transient vibrational spectra of the two NO_2_ product fragments in perfluorohexane. The red lines represent NOα2 which is produced immediately with a thermal population of vibrational states described by a 1350 K Boltzmann distribution. The vibrational populations are simulated to evolve exponentially in time with the relaxation time constants obtained from Fig. 3. The difference between the experimental transient spectra and this simulation are shown as the purple lines, which represent the signal contribution from the NOβ2 fragments. The right hand panel represents the two NO_2_ fragments relaxing to the ground state where the solid green lines are vibrational energy levels of NO_2_. These lines demonstrate how the vibrational levels of NO_2_ become a near continuum above the A state minimum.

The signal contributions from initially generated NOα2 and NOβ2 fragments can be separated using the vibrational relaxation rates obtained from Fig. 3. The NOα2 fragment accounts for the entire signal in the spectral window at Δ*t* < 5 ps, and is generated with a vibrational temperature of 1350 K. This distribution was simulated in the same manner as in the previous section, but instead of optimizing the vibrational temperatures at each Δ*t* to find the best fit, the vibrational temperatures were fixed to relax with the time constants acquired from Fig. 3. The signal arising from the NOβ2 state fragment was then obtained from the difference between the experimental transient absorption signal and this fixed simulation (Fig. 4). The tiny bleach evident at 1610 cm^–1^ in the transient spectra recorded at Δ*t* < 100 ps arises from the 340 nm photolysis of some of the small amount of NO_2_ that is present in the sample in equilibrium with N_2_O_4_. The NOβ2 fragment signal can first be observed after Δ*t* = 5 ps in the PFC solvents, as a localized envelope of absorption signal at the low wavenumber end of the spectral window of Fig. 4 (around the *ν*_3_ = 7 → 8 transition). The NOβ2 fragment signal also appears after Δ*t* = 5 ps in chloroform and CCl_4_, but the broad signal packet rapidly localizes into stretching vibrational modes due to the much faster bend relaxation rates. Transient absorption data showing the signal packet to lower wavenumbers (for higher vibrational levels) in chlorinated solvents are presented in the ESI.‡

From the energy of the 340 nm photon (29 400 cm^–1^), the N–N bond strength in N_2_O_4_ (4500 cm^–1^),^20^ and the average vibrational energy of the NOα2 fragment observed (〈*E*_vib_〉 = 2815 cm^–1^ at 1350 K) the internal energy of the NOβ2 fragment is estimated to be at most 22 000 cm^–1^. This value is consistent with the shortest wavelengths observed from fluorescence measurements of gas-phase N_2_O_4_ photolysis products.^4^ These fluorescence experiments measured an internal energy distribution peaked at approximately 19 000 cm^–1^ for this fragment, corresponding to A^2^B_2_ state NO_2_ (*T*_00_(A–X) = 9710 cm^–1^) born with ∼10 000 cm^–1^ of vibrational energy. The A^2^B_2_ state is strongly coupled to the X^2^A_1_ state which leads to long radiative lifetimes in a collisionless environment, but internally converts to high vibrational levels of the X^2^A_1_ state after only a few collisions in the gas phase.^21^ Gas-phase thermal lensing measurements of excited NO_2_ colliding with atomic gases (and thus only sensitive to V-T energy transfer), and further IR emission measurements,^8^ found that the vibrational relaxation rate of NO_2_ is considerably faster when it contains >10 000 cm^–1^ of vibrational energy. The authors attributed this enhanced energy transfer to larger amplitude vibrations arising from the mixed electronic character of the molecule above the A^2^B_2_ state threshold.

The gas phase picture is consistent with the observations in solution, which imply rapid transfer of >7000 cm^–1^ of vibrational energy to the solvent bath in the first 5 ps after dissociation. The highest X state vibrational level observable in the probe window of Fig. 4 is *ν*(0, 0, 7) with 11 950 cm^–1^ of vibrational energy, just above the A state threshold. NOβ2 further relaxes below the A state threshold populating *ν*(0, 0, 5) by 10 ps, after which considerable diversity in vibrational relaxation timescales is observed between different solvents. The mixing of the X and A electronic states results in a high density of vibrational levels, which favours semi-classical energy transfer dynamics. We propose that both the large amplitude motions associated with vibrational levels with character typical of a polyatomic molecule high in its ground electronic state potential, and the quasi-continuum of NO_2_ vibrational levels, will promote efficient energy transfer through hard collisions. Consequently, the relaxation rates are similar in all of the solvents up to 10 ps, with V-T energy transfer through impulsive interactions providing the dominant energy transfer mechanism in all solvents when NOβ2 has >10 000 cm^–1^ of internal energy. This energy transfer mechanism is the least efficient in CCl_4_, as the appearance of the NOβ2 fragment signal within our observation window is delayed by ∼5 ps compared to the other solvents.

## Conclusions

The detailed relaxation dynamics of both NO_2_ fragments resulting from N_2_O_4_ photolysis in solution have been observed using transient vibrational absorption spectroscopy. The NOβ2 fragment is produced with nearly 20 000 cm^–1^ of internal (vibronic) energy, the first 10 000 cm^–1^ of which is dissipated to the solvent bath *via* impulsive interactions within the first 10 ps (15 ps in CCl_4_) after its formation. Once the internal energy of NOβ2 drops below the A^2^B_2_ state threshold, the relaxation rate slows dramatically and the bending and stretching modes relax independently and through mechanisms which are highly solvent dependent. The *ν*_1_ and *ν*_3_ stretching modes are coupled, and the energy of the high frequency *ν*_3_ mode appears to be funnelled out of the molecule through *ν*_1_ relaxation in most cases.

NO_2_ vibrational relaxation rates in perfluorohexane, perfluoromethylcyclohexane, and perfluorodecalin are nearly identical and are governed by impulsive V-T interactions with the solvent. The relative relaxation rates of the bending and stretching modes of NO_2_ match their relative frequencies, consistent with semiclassical theories of quantized cooling in a classical bath.

Below the A^2^B_2_ state threshold, NO_2_ relaxation through impulsive V-T interactions becomes inefficient in the chlorinated solvents. Vibrational resonances become important, leading to rapid bend relaxation in all three chlorinated solvents, and faster stretch relaxation in chloroform where the C–H bend vibrational frequency is near resonant with the NO_2_ symmetric stretch. The relative relaxation rates of the bending and stretching modes are consistent with expectations from Förster theory, however, rapid cooling of vibrational energy through anharmonic coupling to the chlorinated solvents at room temperature is impeded by solvent heating.

These detailed observations regarding the solvent-dependent relaxation mechanisms of NO_2_ provide a benchmark against which theoretical models can be tested. In turn, these models will provide better understanding of how the excess energy released by exothermic reactions dissipates in a solvent, and whether it influences branching between competing product channels.

## Experimental section

Vibrational transient absorption spectra were collected using the ultrafast laser system at the University of Bristol.^22^ All chemicals were sourced from Sigma-Aldrich. N_2_O_4_ solutions were prepared in perfluorohexane (99%), perfluorodecalin (95%), perfluoromethylcyclohexane (90%), carbon tetrachloride (≥99.9%), chloroform (≥99%), and d-chloroform (99.8% D) by bubbling NO_2_ (≥99.5%) through the pure solvents. The solvents were degassed prior to bubbling by a freeze–pump–thaw method using liquid nitrogen. N_2_O_4_ was photolysed using a 340 nm pump pulse of approximately 120 fs duration and 1 μJ of energy with a repetition frequency of 500 Hz. A broad band (300 cm^–1^) IR pulse was used to probe the samples at 1 kHz and then focused onto a 128 element array detector. The pump–probe time delay was varied using a motorized translation stage while the sample was circulated through a PTFE cell with CaF_2_ windows and a 250 μm path length using a peristaltic pump. The wavelength scales of the transient vibrational absorption spectra were calibrated using a sample of (*E*)-stilbene. Steady state IR spectra were obtained using a Perkin-Elmer Spectrum II ATR-FTIR spectrometer, and steady state UV-vis spectra were acquired using a Thermo Scientific GENYSIS spectrophotometer. Approximate concentrations of N_2_O_4_ were determined to be on the order of 200 mM from steady state UV-vis spectra, using gas-phase absorption cross-sections.^11,23^ Density functional theory and MP2 calculations of the CHCl_3_–NO_2_ and CCl_4_–NO_2_ complexes were performed in Gaussian 09.^24^
