# Supplementary material for: Unravelling the mechanisms of vibrational relaxation in solution
Source: Chem Sci. 2017 Feb 10;8(4):3062–9. doi: 10.1039/c6sc05234g (PMC5380915; doi:10.1039/c6sc05234g)
Supplement: Supplementary file 1 [file SC-008-C6SC05234G-s001.pdf]

Electronic Supplementary Material (ESI) for Chemical Science.
This journal is © The Royal Society of Chemistry 2017

Supplementary Information:

Unraveling the mechanisms of vibrational relaxation in solution

Authors: Michael P. Grubb[a,b], Philip M. Coulter[a], Hugo J. B. Marroux[a], Andrew J. Orr-Ewing[a], Michael N.R. Ashfold[a]

Author Affiliations:

[a]School of Chemistry, University of Bristol, Cantock's Close, Bristol BS8 1TS, UK.

[b]Department of Chemistry, Fort Lewis College, Durango, Colorado 81301, USA.

*Correspondence to: mpgrubb@fortlewis.edu, a.orr-ewing@bristol.ac.uk, mike.ashfold@bristol.ac.uk

1) NO_2 lineshapes in solution

The lineshape of the ν_3 transition is subtly different in each solvent, with regards to the central wavenumber, homogeneous linewidth, and hindering of free rotation (measured by the Q: P/R branch ratio) as presented in Figure 1.[1] Analysis of the lineshapes therefore provides valuable information about the local solvent environment.

Large solute translational energy leads to homogeneous line broadening in transient spectra due to the increased frequency of perturbing interactions with the solvent cage. Furthermore small solute rotation has been shown to be relatively unhindered in perfluorocarbon (PFC) solutions, leading to the observation of significant P and R rotational band structure in the absorption spectrum. Indeed the final equilibrated lineshape of the NO_2 $\nu_3 = 1 \leftarrow 0$ transition cannot be fit with a single Voigt profile. The initial homogeneous linewidth of the NO_2 transitions following N_2O_4 photolysis in perfluorohexane solution is approximately 39 cm^{-1}, and narrows to its final width of 15.6 ± 0.3 cm^{-1} after about 5 ps. This timescale is consistent with previously measured rotational / translational energy relaxation in perfluorohexane.[1]

The lineshapes observed are best fit with a combination of a Lorentzian broadened gas phase simulation of the rotationally structured absorption band of the antisymmetric stretch of NO_2 and a single Lorentzian function at the central transition wavenumber. The gas phase simulation used rotational constants which remained unchanged under vibrational excitation (A = 8.0 cm^{-1}, B = 0.42 cm^{-1}, C = 0.42 cm^{-1}). The NO_2 ν_3 central transition wavenumber is shifted from the observed gas phase value of 1617 cm^{-1} [2] to approximately 1609 cm^{-1} in PFC solvents and 1603 cm^{-1} in chloroform. The magnitude of the observed shift in the central wavenumber is related to the solvent solute interactions. A small change in the shift in centre wavenumber is observed in PFCs, increasing from perfluorohexane to perfluoromethylcyclohexane and perfluorodecalin, in addition to an increase in the relative contribution of the central Lorentzian component for the lineshape. The ratio of the integrated area of the central Lorentzian to the gas phase simulation component can be related to the energetic barrier to rotation of NO_2 provided by the solvent.[1] This barrier quantifies the degree to which the solvent perturbs the rotational motion and subsequently the vibrational spectrum of NO_2. The small observed solvent dependence suggests a minimal increase in van der Waals forces in PFCs consistent with the trend of increasing boiling point from perfluorohexane to perfluorodecalin. [3,4]

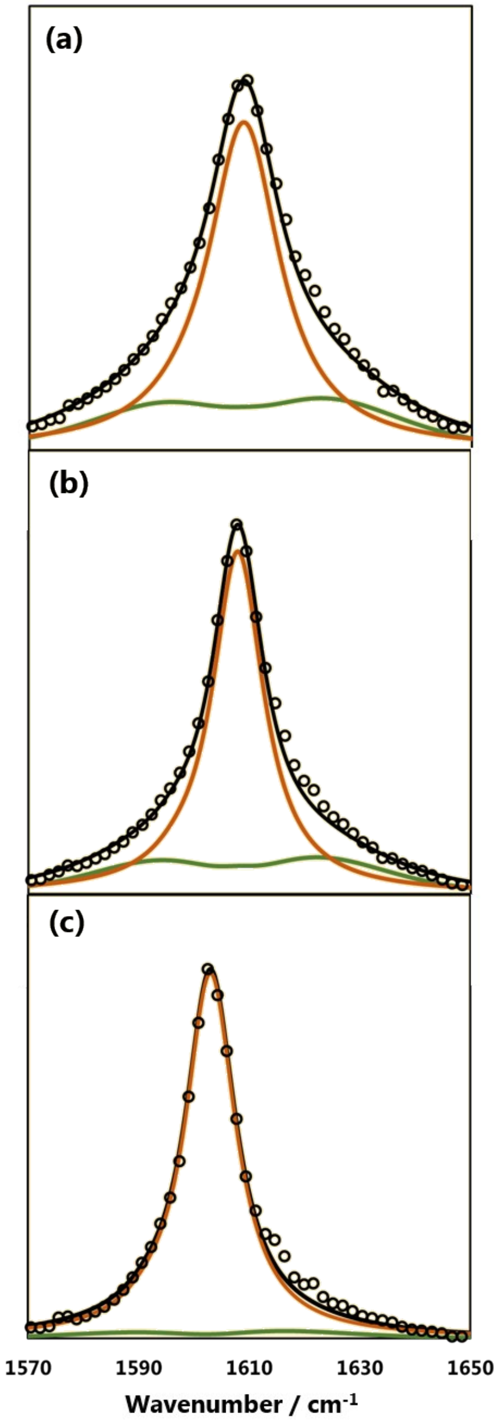

Figure S1: Experimental lineshapes of NO_2 ν_3 absorption (black circles) are fit with a combination (black line) of a single Lorentzian at the centre wavenumber (brown line) and Lorentzian broadened gas phase simulation (green line) of NO_2 ν_3 absorption in (a) perfluorohexane, (b) perfluorodecalin, (c) chloroform.

The Lorentzian full width half maximum (FWHM) observed for the NO_2 ν_3 absorption narrows considerably from perfluorohexane (15.6 ± 0.3 cm^{-1}) to chloroform (11.0 ± 0.2 cm^{-1}). All the absorptions are Lorentzian broadened demonstrating that homogeneous broadening dominates the lineshape. The FWHM of the NO_2 ν_3 absorption observed in chloroform is similar to that observed in perfluorodecalin (11.3 ± 0.3 cm^{-1}) despite a significantly larger central wavenumber

shift and ratio of the central Lorentzian to the gas-phase like component observed in chloroform suggesting that the NO_2 absorption undergoes motional narrowing in chloroform. The increase in FWHM of the NO_2 ν_3 absorption from perfluorodecalin (11.3 ± 0.3 cm^{-1}) to perfluoromethylcyclohexane (14.4 ± 0.4 cm^{-1}) to perfluorohexane (15.6 ± 0.3 cm^{-1}) is more difficult to explain given the similarity of the PFC solvents and their weak interactions with solutes.

2) Transient vibrational absorption (TVA) spectra

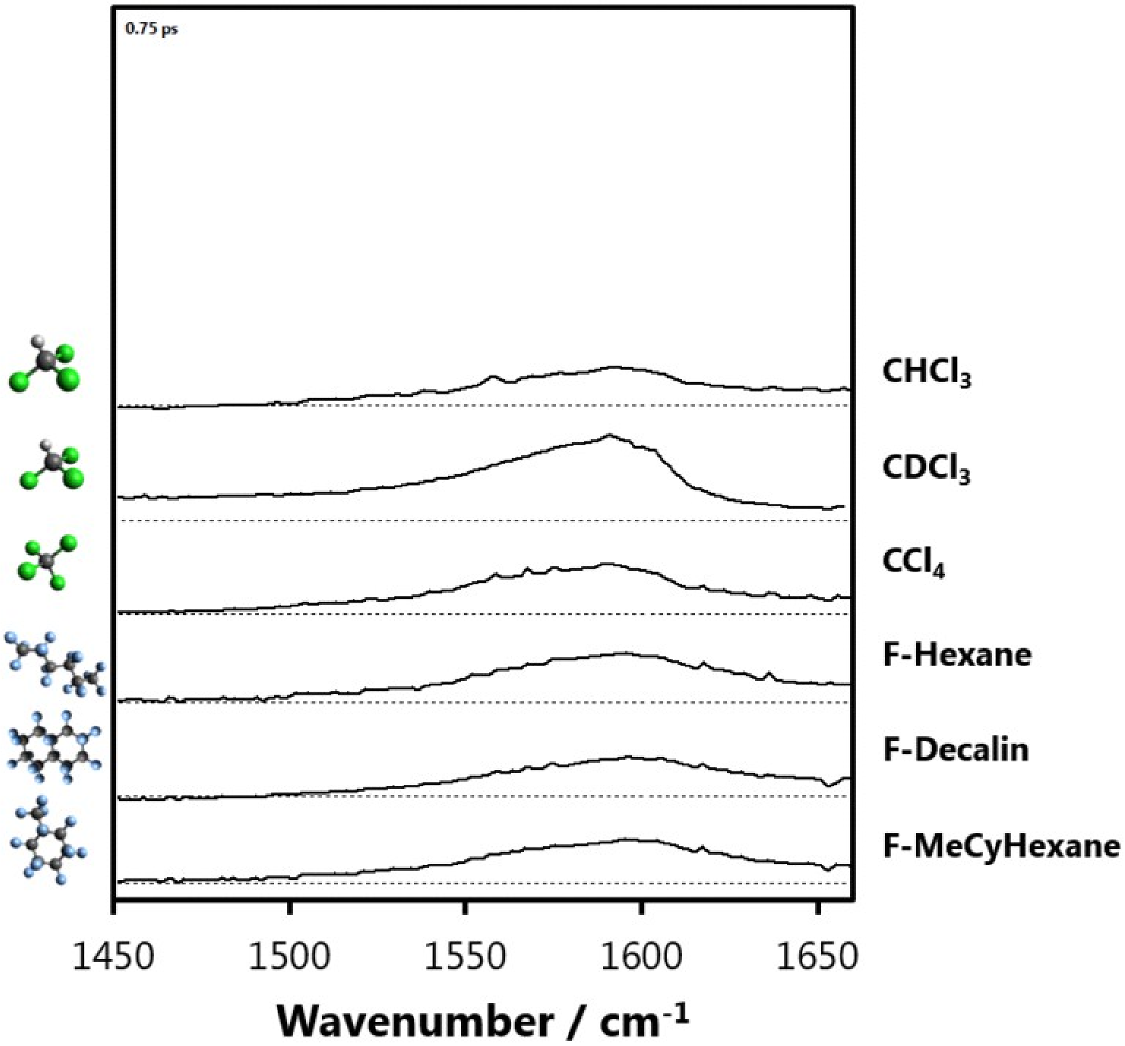

Figure S2: Animation of the time-dependence of the TVA spectra of NO_2 in (from top) chloroform, d-chloroform, carbon tetrachloride, perfluorohexane, perfluorodecalin and perfluoromethylcyclohexane.

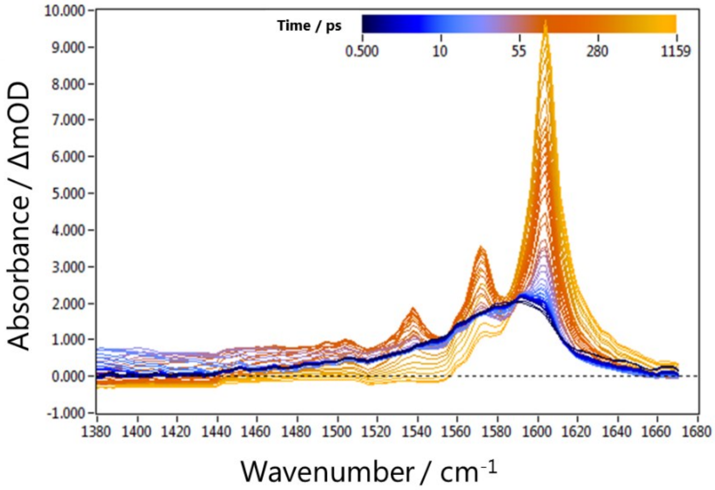

Figure S3: TVA spectra in the NO_2 antisymmetric stretch region following photolysis of N_2O_4 in chloroform.

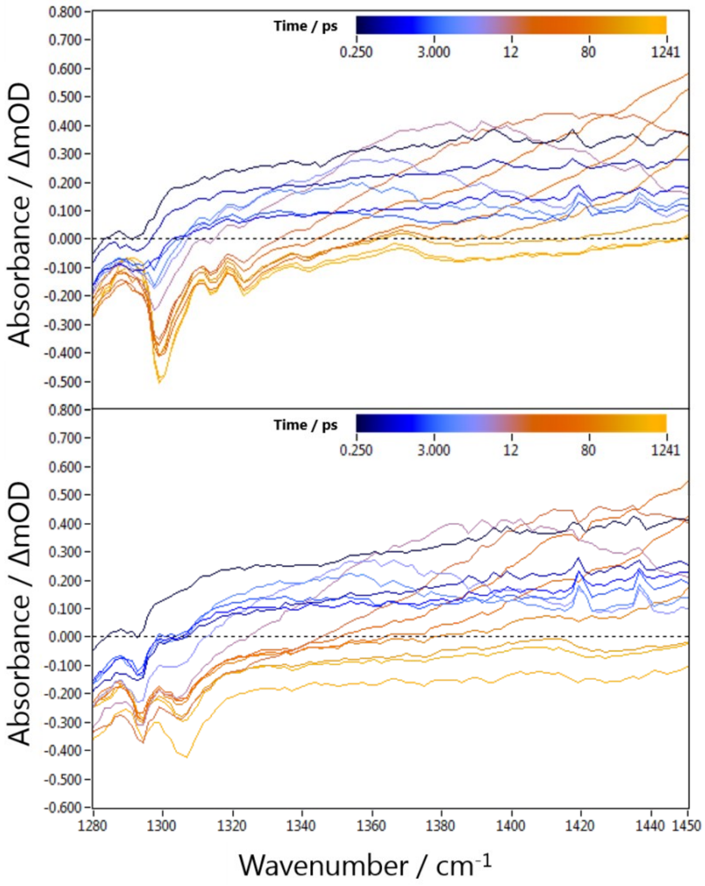

Figure S4: TVA spectra following photolysis of N_2O_4 in $CDCl_3$ (top) and $CHCl_3$ (bottom) showing antisymmetric stretch absorption of highly excited NO_2 as early time (blue) features that evolve to higher wavenumber as the NO_2 cools (orange). After ~300 ps these features have shifted out of the observation window, but can be seen in Figure S2.

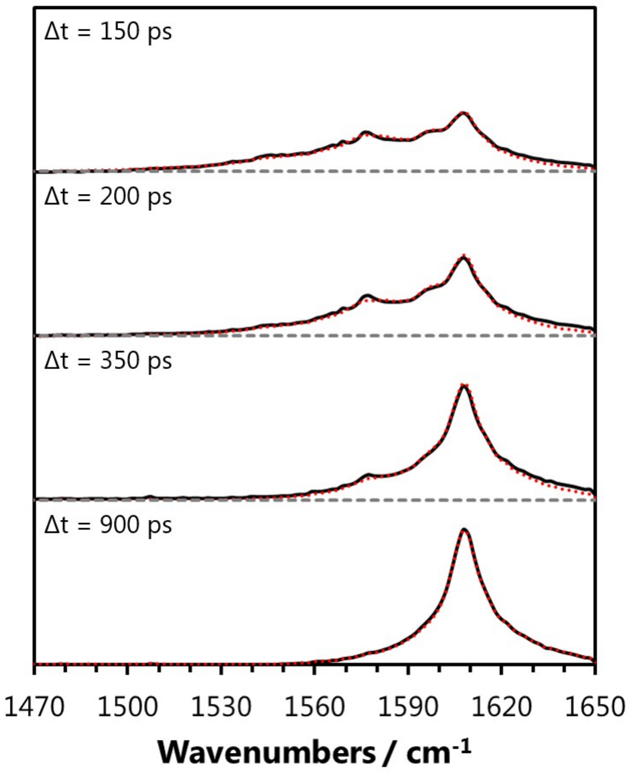

Figure S5: Transient spectra of the antisymmetric stretch of NO_2 produced from N_2O_4 photodissociation at 340 nm in perfluorodecalin solution. The black lines show the experimental spectra which have been fit to a Boltzmann distribution of NO_2 vibrational states shown as the red dotted lines. The total spectral feature can be fit by a single Boltzmann distribution from time delays of 150 ps the temperature of which decays exponentially with time.

3) Concentration dependence of transient data

N_2O_4 exists in the gas phase in equilibrium with NO_2. The N_2O_4 molar dissociation constant (K_c = 0.0060)[5] shows that N_2O_4 is the dominant species in the gas phase at 298 K. The normal molarity (c^o) corresponds to 1 mol dm^{-3}.

$$N_2O_4 \rightleftharpoons 2NO_2$$

$$K_c = \frac{[NO_2]^2}{[N_2O_4]} \frac{1}{c^o}$$

When dissolved in solution the equilibrium constant shifts significantly further in favour of N_2O_4. Values of K_c have previously been determined in chloroform (K_c = 5.3×10^{-6}) and carbon tetrachloride (K_c = 5.9×10^{-6}).[5] The concentration of N_2O_4 can be determined from a given concentration of NO_2 moieties (c_{NO_2}) present using:

$$[N_2O_4] = \frac{4 \times \left({c_{NO_2}}/{c^o} \right) + K_c - \sqrt{8K_c \times \left({c_{NO_2}}/{c^o} \right) + K_c^2}}{8}$$

Concentration dependent transient absorption measurements of N_2O_4 photodissociation were conducted by dilution of saturated solutions to reduce the relative concentration of NO_2 in carbon tetrachloride and perfluorohexane. The growth of the ground state absorption of the

antisymmetric stretch of NO_2 was followed after 100 ps by integrating the high wavenumber side of the ν_3 $(1\leftarrow0)$ transition. The exponential time constant for the growth of the ν_3 $(1\leftarrow0)$ transition was plotted against relative concentration of N_2O_4 (as determined by dilution factors). The linear decrease in time constant with N_2O_4 concentration shown in Figure S6 suggests that vibrational relaxation of NO_2 is somewhat enhanced through interaction with N_2O_4 molecules.

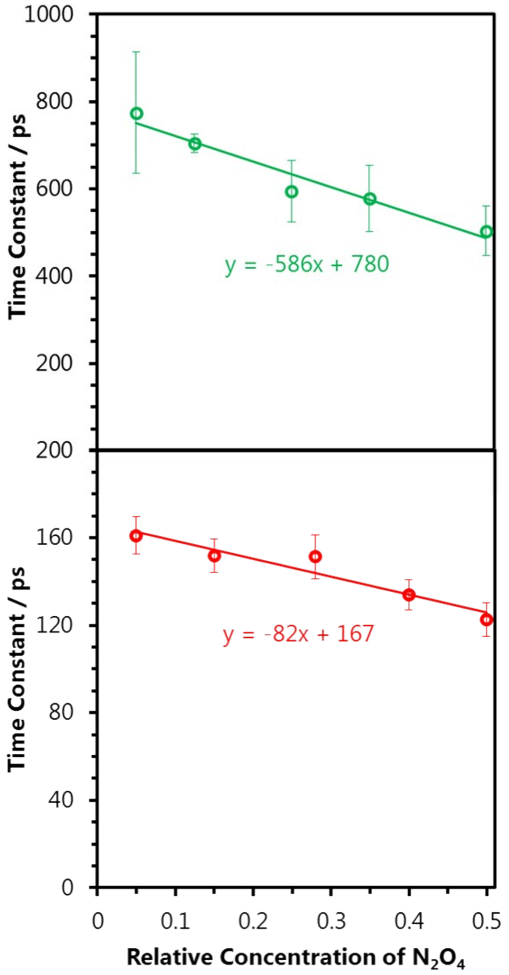

Figure S6. Linear fits to the change in exponential time constants for the growth of the ν_3 $(1\leftarrow0)$ transition of NO_2 produced through 340 nm excitation of N_2O_4 at various relative concentration of N_2O_4 in CCl_4 (green, top panel) and perfluorohexane (red, bottom panel). Error bars are calculated as twice the standard deviation in time constants extracted from integrating the high wavenumber side of the ν_3 $(1\leftarrow0)$ band of NO_2.

The extrapolated time constants at zero concentration (780 ps in CCl_4 and 167 ps in perfluorohexane) result from interaction of NO_2 with solvent molecules only. They identify a much longer relaxation time scale in carbon tetrachloride than in perfluorohexane, consistent with the analysis in the main text. The gradient of the concentration dependence is approximately seven times larger in magnitude in CCl_4 than perfluorohexane. This difference could reflect different diffusion timescales, but is better explained by an approximately 7 times higher saturation concentration of NO_2 in CCl_4.

Similar analysis of the high wavenumber side of the ν_3 $(1\leftarrow0)$ transition in the transient absorption data used to determine thermal cooling time coefficients in the main paper suggests that the N_2O_4 concentrations correspond to points of the graphs in Figure S6 for N_2O_4 relative concentration of approximately 0.25 in perfluorohexane and less than 0.05 in CCl_4. This correlates

well with the expectation of approximately the same N_2O_4 concentration in both solvents given the 7-fold greater solubility in CCl_4. Furthermore, the deviation of the time constant for growth of the ν_3 (1←0) transition at relative N_2O_4 concentrations of 0.25 (PFH) and 0.05 (CCl_4) from zero concentration (~20 ps) is relatively small compared to the vibrational time constants investigated in the main text suggesting that semi-quantitative comparison between the datasets in different solvents is reasonable.

4) IR pump – IR probe data

Transient infrared pump – infrared probe experiments were conducted on the antisymmetric stretch of solvated NO_2. An IR pump pulse centred at 6300 nm vibrationally excited NO_2 ν_3 (1←0) which was then probed by a second equivalent IR pulse. The recovery of the ground state was fit to a bi-exponential (or a single exponential in PFCs). The recovery of ground state NO_2 in $CHCl_3$ was dominated by a 23 ± 2 ps (24 ± 3 ps in $CDCl_3$) time constant, recovering approximately 90 % of the population. A slower time constant on the order of 300 ps was responsible for the remainder of the population recovery. Similarly in CCl_4 a fast recovery (62 ± 10 ps) of 67 % of the population was followed by a recovery with a timescale on the order of a nanosecond.

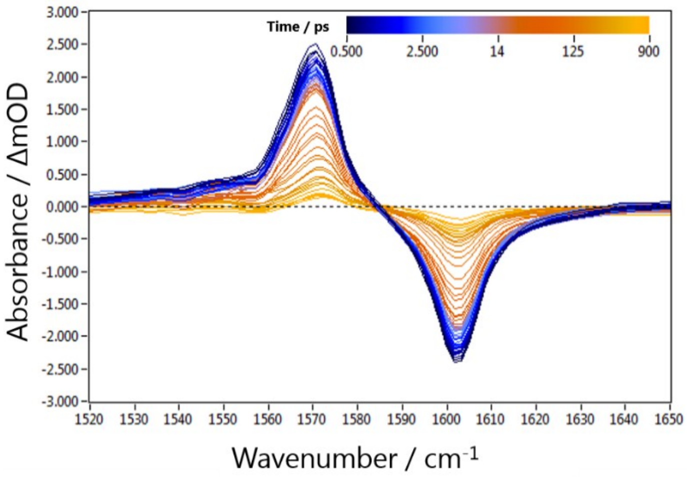

Figure S7. Transient IR-pump IR-probe spectra of the antisymmetric stretch of NO_2 excited to $\nu_3 = 1$ in CCl_4. Spectra are plotted for time delays from 0.5 (blue) to 900 ps (yellow).

The main text explains the faster component of the relaxation of the NO_2 anti-symmetric stretch through anharmonic coupling to the solvent as a result of dipolar interactions. However, if the complexation energy is small, a significant fraction of the NO_2 molecules may not be complexed to the solvent prior to excitation and so are unable to relax their vibrational energy as efficiently, giving the slower relaxation components identified above. The slower time constants are closer to the timescales observed for the growth of the ν_3 (1←0) transition in the UV photolysis of N_2O_4 (> 1 ns for CCl_4; 180 ps for $CDCl_3$; and > 1 ns for $CHCl_3$). If the faster and slower time constants for vibrational relaxation are attributed to complexed and uncomplexed NO_2, the relative populations would reflect the relative binding energies. Subsequently the cooling of the hot NO_2 produced by N_2O_4 photolysis in chlorinated solvents is better represented by the uncomplexed NO_2 relaxation behaviour.

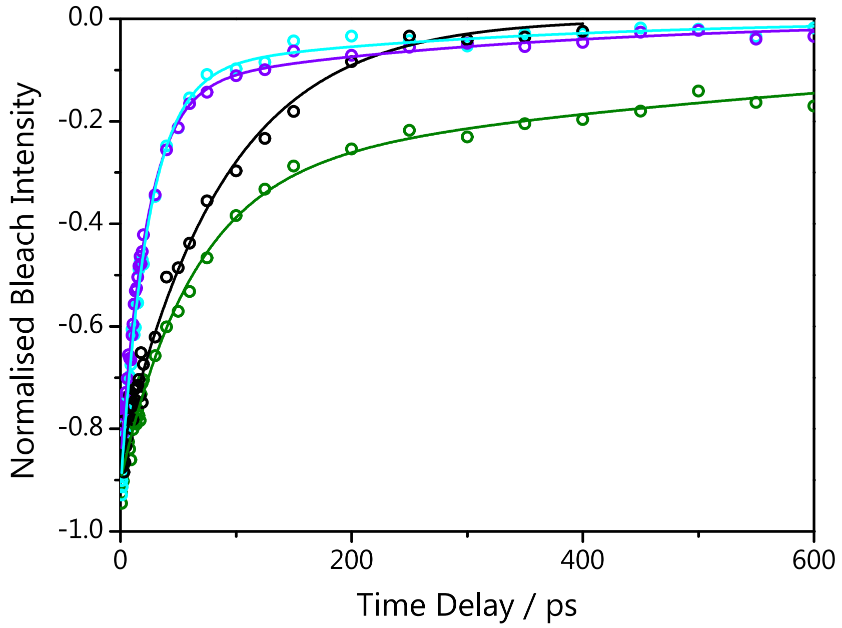

Figure S8. Bleach intensities of the NO_2 antisymmetric stretch band following excitation to $v_3 = 1$, showing time-dependent recovery in CCl_4 (green), perfluorodecalin (black), $CHCl_3$ (purple) and $CDCl_3$ (light blue). The lines are fits to the bleach intensity data (circles) using bi-exponential functions in chlorinated solvents and a single exponential for perfluorodecalin.

In perfluorodecalin the recovery of the ground state NO_2 occurs with a single time constant of 89 ± 7 ps which is similar to the 120 ± 30 ps observed for the growth of the v_3 $(1{\leftarrow}0)$ transition in the UV photolysis of N_2O_4. This outcome is consistent with an absence of dipolar interactions between NO_2 and PFCs, and hence only a weak propensity to form solute-solvent complexes.

5) Binding energy calculations

NO_2 – $CHCl_3$ and NO_2 – CCl_4 complex structures were computed using the Gaussian 09 program.[6] Unrestricted DFT and MP2 methods were used to optimize geometries and calculate binding energies, with account for basis set superposition error using the counterpoise method[7]. Table S1 shows representative values obtained at different levels of theory. The binding energies calculated varied between methods, but are typically smaller than a single quantum of NO_2 antisymmetric stretch vibrational energy, and are smaller for the NO_2 – CCl_4 interaction – consistent with the trend in complexed vs uncomplexed solute ratio implied by the bleach recovery data in section 4 (above).

	WB97XD / 6-311++g(d,p)	CAM-B3LYP / 6-311++g(d,p)	MP2 / 6-311++g(d,p)
CCl_4	315	84	217
$CHCl_3$	633	423	1520

Table S1: Computed binding energies (cm^{-1}) for the interaction of NO_2 with a single CCl_4 or $CHCl_3$ molecule

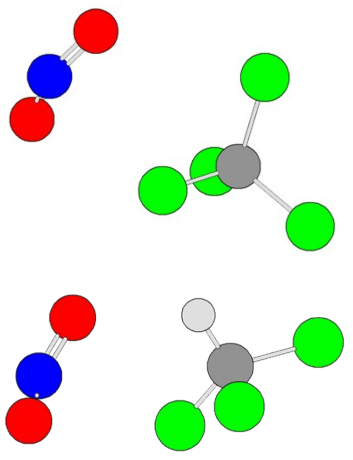

Figure S9: Structures of NO_2 – CCl_4 and NO_2 – $CHCl_3$ complexes optimized at the UMP2 6-311++G(d,p) level of theory.

1. M. P. Grubb, P. M. Coulter, H. J. B. Marroux, B. Hornung, R. S. McMullan, A. J. Orr-Ewing and M. N. R. Ashfold, *Nature Chemistry*, 2016, **8**, 1042-1046.
2. A. Delon and R. Jost, *J Chem Phys*, 1991, **95**, 5686-5700.
3. J. S. Rowlinson and R. Thacker, *Transactions of the Faraday Society*, 1957, **53**, 1-8.
4. A. M. Kunkel, J. J. Seibert, L. J. Elliott, R. Kelley, L. E. Katz and G. A. Pope, *Environmental Science & Technology*, 2006, **40**, 2384-2389.
5. S. N. Mendiara and L. J. Perissinotti, *Appl Magn Reson*, 2003, **25**, 323-346.
6. M. J. Frisch, G. W. Trucks, H. B. Schlegel, G. E. Scuseria, M. A. Robb, J. R. Cheeseman, G. Scalmani, V. Barone, B. Mennucci, G. A. Petersson, H. Nakatsuji, M. Caricato, X. Li, H. P. Hratchian, A. F. Izmaylov, J. Bloino, G. Zheng, J. L. Sonnenberg, M. Hada, M. Ehara, K. Toyota, R. Fukuda, J. Hasegawa, M. Ishida, T. Nakajima, Y. Honda, O. Kitao, H. Nakai, T. Vreven, J. A. Montgomery, Jr., J. E. Peralta, F. Ogliaro, M. Bearpark, J. J. Heyd, E. Brothers, K. N. Kudin, V. N. Staroverov, R. Kobayashi, J. Normand, K. Raghavachari, A. Rendell, J. C. Burant, S. S. Iyengar, J. Tomasi, M. Cossi, N. Rega, J. M. Millam, M. Klene, J. E. Knox, J. B. Cross, V. Bakken, C. Adamo, J. Jaramillo, R. Gomperts, R. E. Stratmann, O. Yazyev, A. J. Austin, R. Cammi, C. Pomelli, J. W. Ochterski, R. L. Martin, K. Morokuma, V. G. Zakrzewski, G. A. Voth, P. Salvador, J. J. Dannenberg, S. Dapprich, A. D. Daniels, Ö. Farkas, J. B. Foresman, J. V. Ortiz, J. Cioslowski and D. J. Fox, *Journal*, 2009.
7. S. F. Boys and F. Bernardi, *Mol Phys*, 1970, **19**, 553.
